# Ultra-Sensitive Piezo-Resistive Sensors Constructed with Reduced Graphene Oxide/Polyolefin Elastomer (RGO/POE) Nanofiber Aerogels

**DOI:** 10.3390/polym11111883

**Published:** 2019-11-14

**Authors:** Weibing Zhong, Haiqing Jiang, Liyan Yang, Ashish Yadav, Xincheng Ding, Yuanli Chen, Mufang Li, Gang Sun, Dong Wang

**Affiliations:** 1 College of Chemistry, Chemical Engineering and Biotechnology, Donghua University, Shanghai 201620, China; weibingzhong09@gmail.com (W.Z.); gysun@ucdavis.edu (G.S.); 2 Hubei Key Laboratory of Advanced Textile Materials & Application, Wuhan Textile University, Wuhan 430200, China; hqjiang@wtu.edu.cn (H.J.); yangliyan0427@163.com (L.Y.); ashish84yadav@gmail.com (A.Y.); dxcooo@163.com (X.D.); chenyuanli2015@126.com (Y.C.); limufang223@126.com (M.L.); 3 Division of Textiles and Clothing, University of California, Davis, CA 95616-8598, USA

**Keywords:** wearable pressure sensor, piezoresistive sensor, fiber assembly, nanofiber aerogel, reduced graphene oxide

## Abstract

Flexible wearable pressure sensors have received extensive attention in recent years because of the promising application potentials in health management, humanoid robots, and human machine interfaces. Among the many sensory performances, the high sensitivity is an essential requirement for the practical use of flexible sensors. Therefore, numerous research studies are devoted to improving the sensitivity of the flexible pressure sensors. The fiber assemblies are recognized as an ideal substrate for a highly sensitive piezoresistive sensor because its three-dimensional porous structure can be easily compressed and can provide high interconnection possibilities of the conductive component. Moreover, it is expected to achieve high sensitivity by raising the porosity of the fiber assemblies. In this paper, the three-dimensional reduced graphene oxide/polyolefin elastomer (RGO/POE) nanofiber composite aerogels were prepared by chemical reducing the graphene oxide (GO)/POE nanofiber composite aerogels, which were obtained by freeze drying the mixture of the GO aqueous solution and the POE nanofiber suspension. It was found that the volumetric shrinkage of thermoplastic POE nanofibers during the reduction process enhanced the compression mechanical strength of the composite aerogel, while decreasing its sensitivity. Therefore, the composite aerogels with varying POE nanofiber usage were prepared to balance the sensitivity and working pressure range. The results indicated that the composite aerogel with POE nanofiber/RGO proportion of 3:3 was the optimal sample, which exhibits high sensitivity (ca. 223 kPa^−1^) and working pressure ranging from 0 to 17.7 kPa. In addition, the composite aerogel showed strong stability when it is either compressed with different frequencies or reversibly compressed and released 5000 times.

## 1. Introduction

Flexible wearable pressure sensors have received extensive attention in recent years because of their benefits such as integratability, their lightweight nature, and their portability [1,2,3,4,5,6,7]. Compared to the capacitive, piezoelectric, and triboelectric sensors, piezoresistive sensors were widely applied in health management, humanoid robots, human machinery, and artificial intelligence due to their simple structure and easily collectable signal [8,9,10,11,12,13,14]. However, the low sensitivity still restricts the practical applications of the piezoresistive sensors. Recently, many structures were designed and constructed to improve the device performance including sensitivity and the working pressure range. Among them, fiber assemblies were considered as ideal substrates that can help improve the sensitivities due to their remarkable deformation ability [15,16,17,18,19,20,21]. When the external pressure was loaded, the porous structures constructed by the stacking fibers present larger deformation when compared to solid materials, which resulted in greater growth of the contacting areas of the conductive components. The increasing interconnection of the conductive components formed more effective conductive networks, which improves the sensitivity of the piezoresistive sensors. In addition, research indicates that a higher porosity of the porous substrates will further strengthen the sensitivity of the piezoresistive sensors [13,22].

Nanofiber aerogels, which were obtained by removing the solvent component from the nanofiber suspension in a supercritical state, possess the highest porosity (>80%) among the fiber assemblies. The randomly distributed POE nanofibers with a high length-diameter ratio would lead to hierarchical self-entanglement and would help form a three-dimensional nanofiber-based network [23,24]. Moreover, the ultra-high specific surface area (>500 m^2^/g) of the nanofiber aerogel provides the structural basis for the interconnection of conductive components under compression. Therefore, a highly sensitive piezoresistive sensor can be expected by using the nanofiber aerogels as the flexible substrate. However, traditional aerogels always show narrow weak compression strength and low structural stability under revised external pressure [25,26,27]. Their internal structure will be easily destroyed under excessive external pressure. As a result, the working pressure range and the operational stability of the nanofiber aerogels-based piezoresistive sensor are generally difficult to meet the requirements of practical use. Literature [28] indicates that the aero carbon materials can enhance the mechanical property by forming the hierarchical three-dimensional structure. Therefore, designing and constructing a three-dimensional carbon material/nanofiber composite aerogel might be an effective solution to achieve ultra-high sensitivity, a wide working pressure range, and excellent cycle stability simultaneously for flexible piezo-resistive sensors.

In the present research, the three-dimensional RGO/POE nanofiber composite aerogels were prepared by chemically reducing the GO/POE nanofiber composite aerogels, which were obtained by freeze drying the mixture of the GO aqueous solution and the POE nanofiber suspension. The RGO/POE nanofiber proportions were adjusted to improve the sensing performance of the composite aerogels. It was found that the volumetric shrinkage of thermoplastic POE nanofibers during the reduction process enhances the compression strength of the composite aerogels while decreasing the sensitivity slightly. Therefore, the sensitivity and working pressure range of the composite aerogel were balanced by adjusting the additional amount of the POE nanofibers. The results indicate that the composite aerogel with POE nanofiber/RGO proportion of 3:3 was the optimal sample, which exhibits high sensitivity (ca. 223 kPa^−1^), wide working pressure range (0–17.7 kPa), and strong stability either compressed with different frequencies or reversibly compressed and released for 5000 times.

## 2. Materials and Methods

Polyolefin elastomer (POE) and cellulose acetate butyrate (CAB. Butyrate content 35–39%) were purchased from Sigma-Aldrich (Saint Louis, MO, USA), Dow Chemical Company (Midland, MI, USA) and Acros Chemical Co. Ltd., Geel, Belgium, respectively. Tertiary butanol, acetone, concentrated sulfuric acid, potassium permanganate, and hydrochloric acid were from Sinopharm Chemical Reagent Co., Ltd., Shanghai, China. The dispersing agent was supplied by Lubrizol (Lake County, OH, USA). Deionized water is self-made in the laboratory. All the chemicals are used without further purification. The micro morphologies were observed by JSM-6510LV (JEOL, Tokyo, Japan). The chemical structures were measured via FTIR mechine of Tensor 27 (Bruck, Karlsruhe, Germany) The resistances of the aerogels were measured via the 15b+ multimeter (Fluke, Washington, USA). The I-t characteristics of the pressure sensors were collected through ST600L motorized dynamic resistance station (Shente, Shanghai, China) 

The POE nanofibers were prepared by extraction removal of the CAB from the POE/CAB composite fiber obtained sea island method. Previous studies reported the detailed method [7,29,30,31,32]. Then, the POE nanofibers were dispersed in tertiary butanol-water and the dispersing agent under high speed (10,000 r/m) shearing. The dispersing agent marked with Lubrizol 27,000 was used to help the uniform dispersion process of the POE nanofiber with a 10% mass proportion. The suspension was purified via a filter with a diameter of 150 μm to remove the aggregations. The photographs of the obtained POE nanofiber were presented in Figure S1, which indicates the good uniformity.

**Figure 1:**
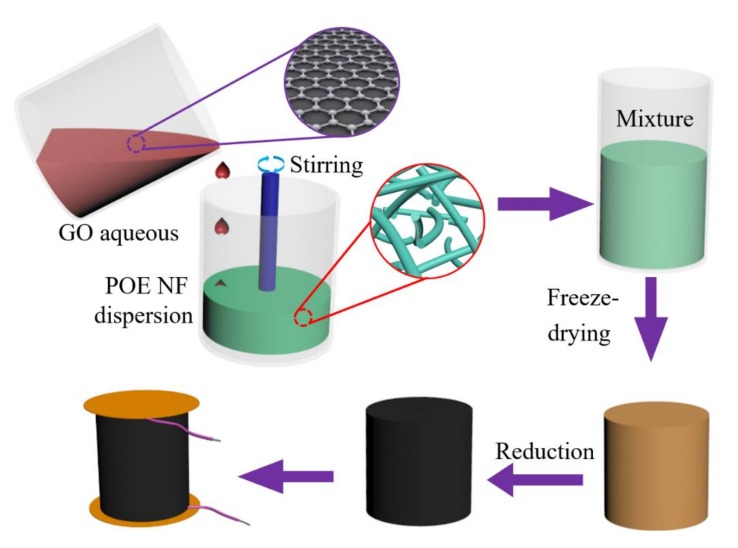
The illustration schematic of the preparation of the RGO/POE nanofiber composite aerogels.

The GO aqueous solution was prepared using a modified Hummer’s method. The concentration using GO aqueous solution was 5 mg/mL. As shown in Figure 1, the prepared POE nanofiber suspension and the GO aqueous solution were directly mixed together by continuously stirring, according to a GO/POE nanofiber proportion of 6:0, 5:1, 4:2, 3:3, 2:4, and 0:6, respectively. After 5 min of ultrasonic treatment, the mixtures were transferred to a low temperature freezer (−38 °C) for 8 h. The GO/POE nanofiber aerogels were obtained after the mixtures were freeze dried for more than 24 h.

A total of 10 mL of hydrazine hydrate aqueous solution was added to the bottom of the beaker. The obtained GO/POE nanofiber aerogels were placed on a suspension bracket hanging in the baker. Then the beaker was placed in an oven at 90 °C for 100 min after the beaker was sealed. The RGO/POE nanofiber aerogels were obtained after they were placed in a fume hood for more than 6 h.

## 3. Results and Discussions

**Figure 2:**
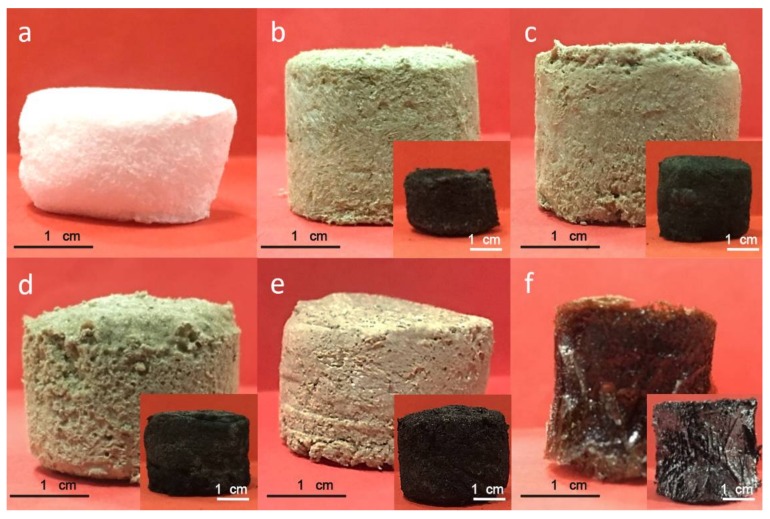
The photographs of the composite aerogels with POE nanofiber/GO proportion of (**a**) 6:0, (**b**) 5:1, (**c**) 4:2, (**d**) 3:3, (**e**) 2:4, and (**f**) 0:6. The inserted images were the photographs of corresponding aerogels after being reduced.

The photographs of the prepared aerogels with a different proportion of the POE nanofiber/RGO were shown in Figure 2. As shown, the aerogel containing GO could favorably maintain the cylindrical shape after demolding, and it showed the same maple color as the pure GO aqueous solution. After air phase reduction by a hydrazine vapor, the reduced aerogels were all converted to a black color. The aerogel had a volume retraction during the reduction process. The shape retention rate of the aerogel during the reduction process was shown in Table S1. The aerogel with more POE content resulted in the lower shape retention rate. The lowest shape retention rate at only 14.91% appeared on the aerogel with a POE nanofiber/GO proportion of 5:1. This was caused by the softening and heat-induced shrinkage of the POE nanofibers under the high temperature (90 °C) during the reduction process. The shrinkage during this process may contribute to the physical crosslinking between the softening POE nanofibers, which helps improve the structural stability of the aerogels. The volume of the pure RGO aerogel reached 140.48% of the GO aerogel. This might be caused by the straightening of curled GO after undergoing a reduction. However, it can be easily observed that the pure GO aerogel had lamellar structures and the flake graphene is prone to chipping and slag. The lamellar structure easily collapsed under pressure.

**Figure 3:**
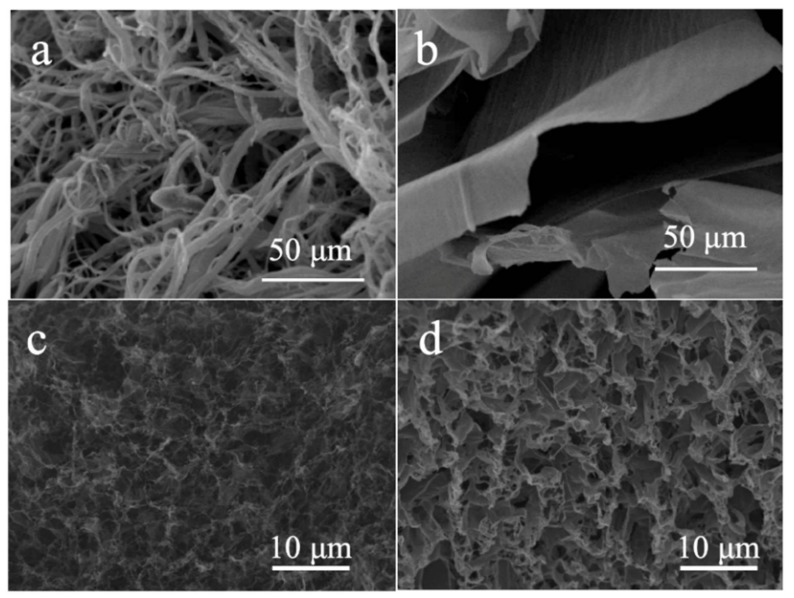
The SEM images of aerogels comprised of (**a**) pure POE nanofibers, (**b**) pure RGO, (**c**) GO/POE nanofibers, and (**d**) RGO/POE nanofibers.

The microstructure of the prepared aerogel was observed by scanning electron fiber microscopy (SEM). Figure 3 presented the SEM of pure POE nanofiber aerogel, pure RGO aerogel, GO/POE nanofibers aerogel, and RGO/POE nanofibers aerogel (POE: GO = 3:3). As observed, the pure POE nanofiber aerogel was composed of fluffy nanofibers, which were randomly distributed. In the pure RGO aerogel, the stacking structure of reduced RGO sheets with wrinkles on the surfaces can be easily observed. There is no nanofiber support between the sheets, which may result in the collapsing structure of the pure RGO aerogels. For the composite aerogels of GO/POE and RGO/POE nanofiber aerogels, the uniform porous structures were constructed since the graphene and the fibers were intertwined. As can be seen, the GO layer was extremely thin in GO/POE nanofiber aerogels, which made it look like a semi-transparent substance. After undergoing a reduction, the pore size decreased and the structure became compact, which may be induced by the volumetric shrinkage during the reduction process.

**Figure 4:**
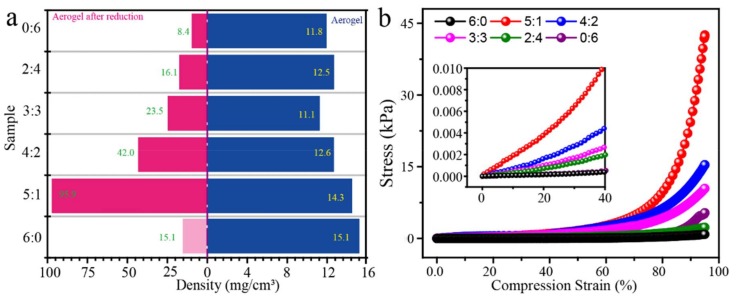
(**a**) The density of the prepared aerogels before and after a reduction, (**b**) the compression stress and strain curve of the prepared aerogels with different POE nanofiber/RGO proportions.

Figure 4 showed the density of the aerogel material before and after reduction and the compressive stress and strain curves of the aerogels after undergoing a reduction. It can be seen from Figure 4a that the density of the GO/POE nanofiber aerogel before reduction was relatively low, which distributed between 11.1–15.1 mg/cm^3^. The density of the pure RGO aerogel decreased from 11.8 to 8.4 mg/cm^3^ after undergoing a reduction. For the RGO/POE nanofiber composite aerogels, the density increased to some degree. The aerogels with more POE nanofibers content exhibited a greater density increase. Among them, the highest density reached 95.9 mg/cm^3^. It is mainly related to the volumetric shrinkage during the reduction process. Since the aerogel before a reduction is easily collapsed and the compression test cannot be performed, Figure 4b only showed the compressive stress and strain diagram of the aerogel after undergoing a reduction. It can be seen that the compressive stress of aerogel increased very slowly in the low strain region but enhanced rapidly in the high strain region. This may be due to the high porosity of the aerogel, which requires a large amount of strain space to squeeze out the internal air. In addition, compared with the aerogels with different RGO/POE proportions, the aerogels with more POE nanofiber content have higher stress at the same strain, which represents stronger compression strength. This may be caused by the higher density and solid content that induced more force units during compression in aerogels with more POE nanofibers content, which makes it easier to generate greater stress.

**Figure 5:**
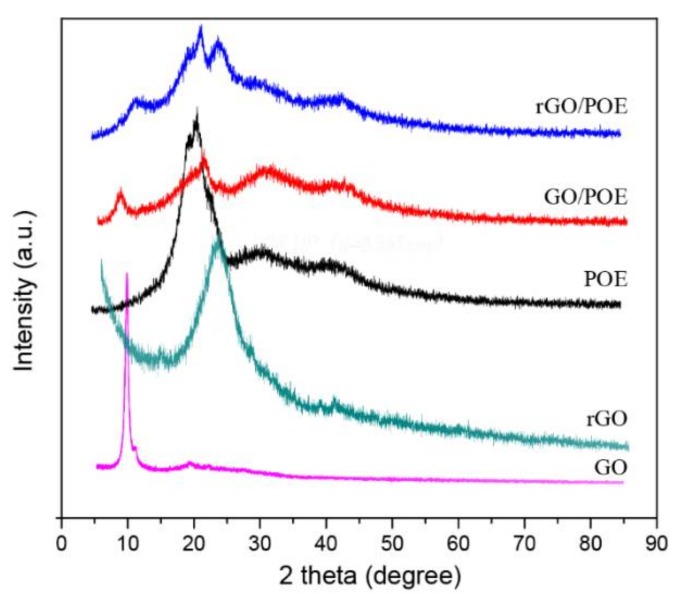
XRD spectrum of the prepared aerogels composed by GO, RGO, and POE nanofiber, GO/POE, and an RGO/POE nanofiber composite aerogel.

Figure 5 presented the XRD spectra of the prepared aerogel materials of pure GO and RGO. It can be seen that the peak of pure GO and RGO appeared at 9.92° and 24.10°, which means that the spacing of graphene sheets is 0.891 nm and 0.368 nm, respectively. Since the POE is partially crystallized, which acted as the physical crosslinking section, the POE exhibits several characteristic peaks. As shown, the peak position of pure POE appeared at 19.81°, 20.93°, 30.72°, and 41.64°. When the GO is added into the aerogel, the peak position of GO blue shifted (2θ = 8.50°), which suggested that the addition of POE leads to a larger spacing of the GO layer. The space reached 1.02 nm from 0.891 nm, which indicates that the POE nanofiber was uniformly mixed. In the RGO/POE nanofiber aerogel, the peak (2θ = 24.10°) is the same as pure RGO. Therefore, the mixing of POE and GO forms an impedance effect on the GO graphitization process, which results in undiminished RGO layer spacing.

**Figure 6:**
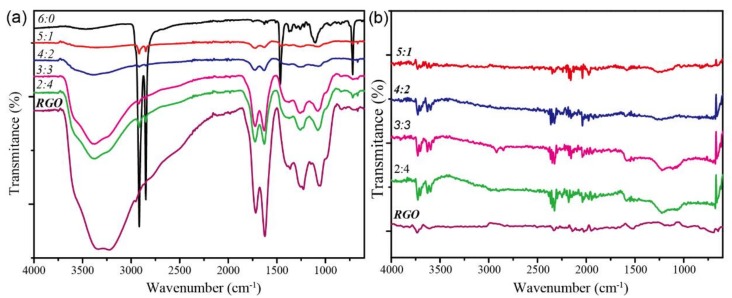
The FT-IR spectrums of the prepared aerogels with different POE nanofiber/RGO proportions (**a**) before and (**b**) after undergoing a reduction.

The FTIR of the prepared aerogel before and after a reduction were measured in Figure 6. The absorption peaks at 3300, 1718, 1625, 1230, and 1066 cm^−1^ represent the free hydroxyl bonds, carbon-oxygen double bonds, and epoxy groups on the carbonyl group, respectively. The absorptions were complex in the 1300 cm^−1^ to 400^−1^ region, which may include the carbon-oxygen stretching vibration, stretching resonance of carbon-sulfur bonds, and skeleton vibration. The absorption peaks of POE mainly at 2914, 2834, 1463, 1102, and 713 cm^−1^ represented the stretching resonance of the single bond in the methylene group and the in-plane and out-of-plane bending resonance absorption peaks of carbon-hydrogen bonds, respectively. It is noticeable that, with the decrease of GO content, the peak intensity of GO significantly decreased, while the absorption peak of POE gradually appeared. Moreover, even when the mixed ratio of POE and GO reached 5:1, the characteristic absorption peaks of POE were still weak. It can be inferred that the two-dimensional GO uniformly encapsulated the POE nanofibers, which makes it difficult to obtain the absorption peak of POE by using the measurement method of surface reflection. From Figure 6b, only extremely weak characteristic absorption peaks at 3729, 3630, 2328, and 1213 cm^−1^ remained after the reduction while most of the other absorption peaks disappeared. It suggested that there were rare free hydroxyl groups and a small amount of triple bonds or cumulative double bonds, which indicates a higher degree of reduction. Moreover, the few active groups mean the relatively stable chemical properties of the composite aerogels, which may make it stronger in the anti-interference ability in practical uses.

**Figure 7:**
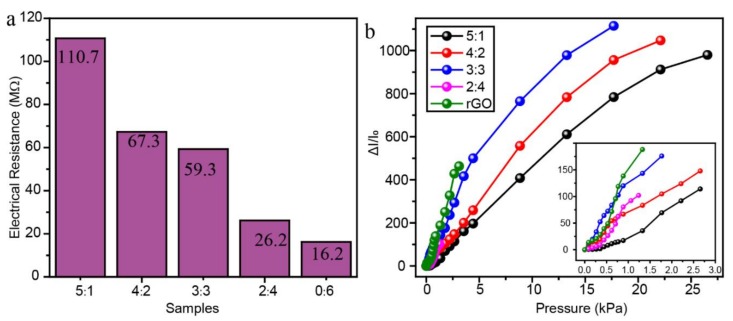
(**a**) The electrical resistance and (**b**) the relative current change curves under different pressures of the prepared composite aerogels with different POE nanofiber/RGO proportions.

The electrical resistance and relative current changes under different pressures of the RGO/POE nanofiber aerogels with a different RGO/POE proportion were measured and the results were shown in Figure 7. Because of the low solid content of the aerogels, the conductive RGO sheets were difficult to interconnect into an effective conductive network. Therefore, the aerogels exhibited very high electrical resistance of ca. 10^7^–10^8^ Ω (Figure 7a). The electrical resistance of the aerogel decreased when the amount of RGO increased. The aerogel with the POE/RGO proportion of 5:1 reached the highest electrical resistance of ca. 110.7 MΩ while the pure RGO aerogel shows the lowest resistance of ca. 16.2 MΩ. Figure 7b presented the ΔI/I_0_ versus pressure curves of aerogels with different RGO/POE nanofiber proportions. As shown, the obtained aerogel samples all exhibited an increasing relative current change (ΔI/I_0_) when exposed to raising external pressures. The more RGO usage led to a higher ΔI/I_0_ value but narrower working pressure ranges, which were determined by varying inner conductive networks and compression mechanical properties. Aerogels with more RGO usage present higher possibilities to form extra interconnections of conductive RGO sheets. Meanwhile, the aerogel with the higher RGO amount possess a lower density and smaller stress under the same compression deformation, according to the results in Figure 4.

**Figure 8:**
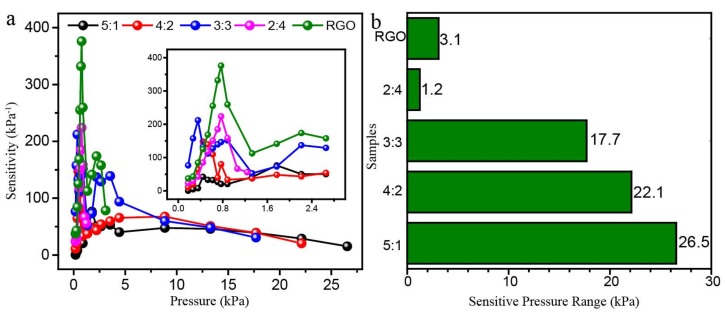
(**a**) The sensitivities and (**b**) the working pressure ranges of the prepared composite aerogels with different POE nanofiber/RGO proportions.

The sensitivities of the aerogels were calculated via the definition equation of S = ∂(ΔI/I_0_)/ ∂p. The results were shown in Figure 8a. As can be observed, the prepared aerogel exhibits high sensitivities in the low-pressure region (<5 kPa) and the peaks appeared at the pressure region of 0.5 to 1.0 kPa. The pure RGO aerogel showed the highest sensitivity of 391 kPa^−1^ at a pressure of ca. 0.8 kPa. The highest sensitivity of each aerogel decreased as the RGO usage reduced. The aerogel with POE/RGO proportion of 5:1 exhibited the lowest sensitivity of ca. 83 kPa^−1^. The RGO/POE nanofiber composite aerogels exhibited different working pressure ranges, which was displayed in Figure 8b. As presented, the RGO/POE nanofiber composite aerogels with more POE content possess a larger working pressure range. The aerogel with POE/RGO proportion of 5:1 shows the widest working pressure range of 0–26.5 kPa while the narrowest was from 0–1.2 kPa when the POE/RGO proportion was 2:4. This correlated with the compression mechanical properties of the composite aerogels with different RGO/POE proportions. Taking the sensitivity and working pressure range into consideration, the composite aerogel with the RGO/POE nanofiber was selected as the optimal samples due to the high sensitivity of ca. 223 kPa^−1^ and a wide working pressure range of 0–17.7 kPa. The optimal sample was applied for the following stability testing.

**Figure 9:**
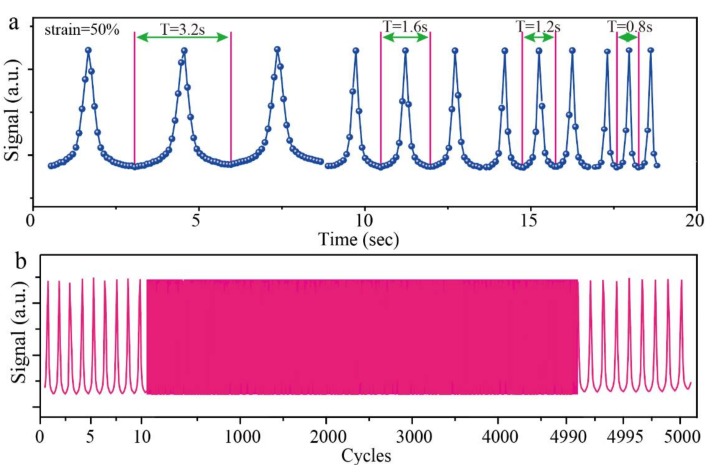
The stability of the prepared composite aerogel with POE nanofiber/RGO proportions of 3:3 under (**a**) dynamic pressure with a different frequency and (**b**) reversible compression and releasing for about 5000 times.

**Figure 10:**
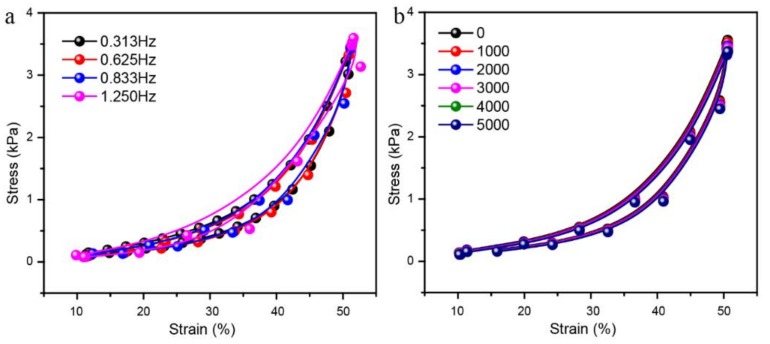
The relative compression stress and strain curve of the prepared sensor under (**a**) a different compression frequency and (**b**) a 5000 times cycling test.

**Figure 11:**
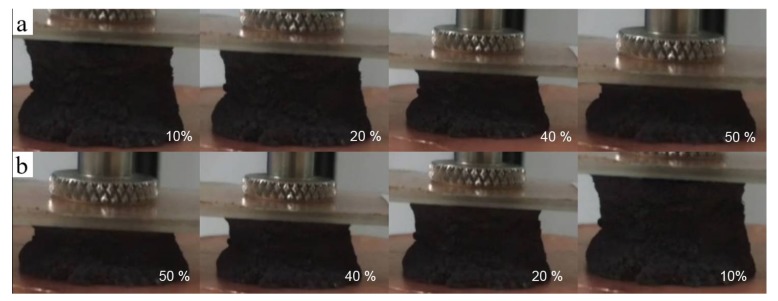
The digital camera photographs of the (**a**) compressing and (**b**) releasing process of the prepared aerogels.

The stability of the composite aerogel with POE/RGO proportion of 3:3 was tested by reversibly applying and removing the compression strain with various frequencies. Due to the high moving speed of the indenter during the stability test, especially in the cycle stability test, the inertia causes a separation of the electrode and the aerogel while the indenter is on the highest point. This phenomenon leads to an inaccurate stability result. Therefore, the compression strain region of 10% to 50% was selected instead of a region between 0% to 50%. The results were presented in Figure 9a. The different compression and releasing periods of 3.2 s, 1.6 s, 1.2 s, and 0.8 s were implemented, respectively. As shown, the signal outputs were regularly arranged upward peaks. The shapes and the values of the signal outputs were almost the same under different compression frequencies, which indicated the good stability of the composite aerogel sensor. In addition, the composite aerogel sensor was repeatedly compressed and released under the strain from 10% to 50% for about 5000 times and the signal output was presented in Figure 9b. As observed, the shapes of the signal outputs remain the same from the beginning to the end of the test. During the 5000 times testing, only the values of the peak tops fluctuate slightly, which suggested good cycling stability of the composite aerogel. Moreover, the compression stress and strain curves under different compressing frequencies and different cycles during the 5000 times cycling tests were displayed in Figure 10a,b. It can be found that only a small amount of stress drawdowns occurred in different frequency compression tests and cycling tests. This further proves the compressive mechanical stability of the RGO/POE nanofiber composite aerogel, which may result in physical crosslinking by the softening of the POE nanofibers during the reduction process. Figure 11 presented several screenshots of the compression and releasing process from a cycling testing video, which suggested that there is no irreversible deformation and almost no reply hysteresis during the whole compressing and releasing process.

## 4. Conclusions

In summary, the three-dimensional composite aerogels were prepared with one-dimensional POE nanofibers and two-dimensional RGO sheets. The RGO/POE nanofiber proportions were adjusted to improve the sensing performance of the composite aerogels. The results suggest that the proportions show great impact on volume shrinkage during reduction, compression mechanical behavior, sensitivity, and a working pressure range of the composite aerogels. The aerogels with more POE nanofiber content exhibit lower volume retention, larger stress under the same compression strain, lower sensitivity, and a wider working pressure range. Taking the sensitivity and the working pressure range into consideration, the composite aerogel with POE nanofiber/RGO proportion of 3:3 was selected as the optimal sample, which exhibits high sensitivity (ca. 223 kPa^−1^) and a wide working pressure range (0–17.7 kPa). It also showed excellent operational stability either compressed with different frequencies or reversibly compressed and released for 5000 times.

## Supplementary Materials

The following are available online at https://www.mdpi.com/2073-4360/11/11/1883/s1, Figure S1: The photographs of the uniform POE nanofiber suspension obtained from different observing angles, Table S1: The volume retention of the prepared GO/POE nanofiber aerogels after reduction.
