# Supplementary material for: Ultra-Sensitive Piezo-Resistive Sensors Constructed with Reduced Graphene Oxide/Polyolefin Elastomer (RGO/POE) Nanofiber Aerogels
Source: Polymers (Basel). 2019 Nov 14;11(11):1883. doi: 10.3390/polym11111883 (PMC6918190; doi:10.3390/polym11111883)
Supplement: Supplementary file 1 [file polymers-11-01883-s001.pdf]

Ultra-Sensitive Piezo-Resistive Sensors Constructed with Reduced Graphene Oxide/Polyolefin Elastomer (RGO/POE) Nanofiber Aerogels

Weibing Zhong[a], Haiqing Jiang[b], Liyan Yang[b], Ashish Yadav[b], Xincheng Ding[b], Yuanli Chen[b], Mufang Li[b], Gang Sun[a,c], Dong Wang[a,b*]

a. College of Chemistry, Chemical Engineering and Biotechnology, Donghua University, Shanghai 201620, China

b. Hubei Key Laboratory of Advanced Textile Materials & Application, Wuhan Textile University, Wuhan 430200, China

c. University of California, Davis, CA 95616-8598 USA

* Corresponding author: wangdon08@126.com (D. Wang)

Table S1. The volume retention of the prepared GO/POE nanofiber aerogels after reduction

POE / GO proportion	5:1	4:2	3:3	2:4	0:6
Volume retention (%)	14.91	30.00	47.23	77.64	140.48

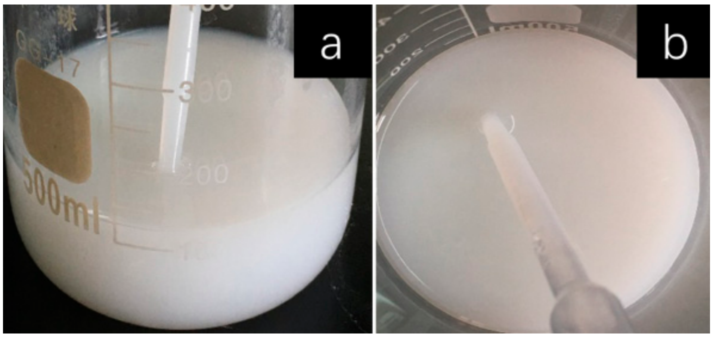

Figure S1. The photographs of the uniform POE nanofiber suspension obtained from different observing angles
